# The thermoregulation of healthy individuals, overweight–obese, and diabetic from the plantar skin thermogram: a clue to predict the diabetic foot

**DOI:** 10.1080/2000625X.2017.1361298

**Published:** 2017-08-16

**Authors:** Francisco-J Renero-C

**Affiliations:** ^a^ Department of Optics & Bio-Medicine, INAOE, Pue, Mexico

**Keywords:** Thermoregulation, thermogram, overweight-obese, diabetic, angiosome, diabetic foot

## Abstract

**Background**: Thermoregulation is a complex autonomic process to keep or to dissipate heat in the human body.

**Methods**: In this work, by means of the thermogram of the plantar skin, the thermoregulation of healthy individuals, overweight–obese, and diabetic is discussed.

**Results**: The thermograms of the plantar skin, for the healthy individuals, are: (1) symmetrical, the temperature distribution of the right foot being a mirror image of that of the left foot ; (2) the thermograms of women, on average, are 3°C colder than those of the men; and (3) the temperature distributions decrease distally from the medial longitudinal arch. The plantar skin thermograms of overweight–obese individuals show: (1) increased average temperature of both feet and for both genders; (2) no symmetry between the left and right feet thermograms; and (3) the temperature distribution is still decreasing from the medial longitudinal arch to the periphery of the foot. However, the standard deviation, for each averaged temperature of the angiosomes, shows greater uncertainty. Most thermograms of diabetic individuals show temperature increase on the plantar skin, and are mostly symmetric between left and right feet.

**Conclusions:** An asymmetric thermogram of the plantar skin of diabetic individuals, where one foot is hotter than the other, may mean that the coldest foot is losing the capacity to communicate properly with the central nervous system and/or that vasoconstriction/vasodilatation is having problems in regulating the passing of blood through the vessels. Thus, the asymmetric thermograms of diabetic patients, and particularly those coldest regions of foot are of interest, because of the reduction of the local autonomic sensing and the lack of achieving properly the passing of the blood.

## Introduction

Thermoregulation is the capacity of humans to control their body temperature within certain limits, even if the surrounding temperature varies considerably. It is part of the autonomic central nervous system (CNU) that integrates, via the thermal sensors, temperature from organs and from the environment, so as to maintain the proper temperature of the body or of the organs. The hypothalamus is responsible for integrating the thermal information from the different sensors. Thus, thermoregulation is accomplished by a complex system from the hypothalamus, the hypophysis, the excretory glands, the peripheral nerve system, and up to the different effectors to keep or to dissipate heat [1]. Thus, one way to keep or to dissipate the heat, in or from, the human body is by the contraction or dilatation of the vessels on the limbs, respectively. Then, the temperature distribution on the limb’s skin varies depending on whether the body is keeping or dissipating heat.

Thermoregulation, in humans, has been investigated by several research groups; for instance, Charkoudian [2] and Tansey et al. [3] reviewed its autonomic mechanisms. Schlader et al. [4] described the thermoregulatory behavior during rest and the exercise. Rutkove et al. [5] studied the thermoregulation of the inferior limbs in diabetic patients, while Houghton [6] tried to prove if the increase in the plantar skin temperature may predict the neuropathic ulceration. The changes of the foot temperature in healthy people have been studied as a function of the ambient temperature and age [7].

From the technological point of view, the temperature distribution of a given object can be studied from the thermogram. The device that registers the thermogram collects the thermal radiation from the study object. In other words, the thermogram is a color false picture of the temperature distribution of the study objects [8].

In this work, the thermoregulation of individuals with no sign of sickness and diabetic patients is described from the thermogram of the plantar skin. Since obesity is not considered as a sickness, but is associated with the risk of developing metabolic disorders [9,10], the thermograms of the individuals with no sign of sickness are separated into two groups based on their body mass index (BMI). Most research on the thermograms of diabetic patients has been done so as to classify the morphology of the temperature distribution [11,12]; here the temperature distributions on the plantar skin are described in terms of its thermoregulation.

Three characteristics emerged from the thermograms of volunteers with BMI < 25 kg/m^2^: (1) symmetry, the temperature distribution on the right foot is a mirror image of that of the left foot; (2) thermograms of women can be distinguished from those of men because the average temperature on the medial longitudinal arch is 26.2 ± 0.9°C and 29.0 ± 0.6°C for women and men, respectively; also, the medial longitudinal arch is the hottest part of the plantar skin for both genders; and (3) the temperature distribution decreases distally from the medial longitudinal arch.

On the other hand, it is not possible to distinguish the gender from the thermograms of individuals who are overweight or obese (BMI ≥ 25 kg/m^2^), since the average temperature of the medial longitudinal arch on the women’s thermograms increased by 3°C, so that they looked like the thermograms of men. Furthermore, the average temperature of the plantar skin, on both feet, increased for women and men. Hence, the temperature of the medial longitudinal arch increased to 29.4 ± 1.1°C and 30.0 ± 1.1°C for women and men, respectively. The symmetry of the thermograms, between the left and right feet, is still observed, and the temperature distribution is still decreasing from the medial longitudinal arch to the periphery of the foot. However, the standard deviations of the average temperature for each of the angiosomes^1^ ^1^An angiosome is a territory of a composite tissue for a given artery (or branches of different arteries), and it is represented on different parts of the skin [14,15]. show greater uncertainty.

The thermograms of the diabetic patients differ a lot from those of the volunteers with no sign of sickness. The most common pattern in the thermograms of the plantar skin of diabetic patients is an increment of the temperature on a symmetric distribution on both feet [12]. However, there are thermograms with asymmetric temperature distribution within the one foot and between the feet. A cold part on the plantar skin, in an asymmetric temperature distribution, means that less blood is passing through the vessels on that part of the skin, which may reflect a deterioration of the autonomic peripheral nervous or malfunctioning of the constriction or dilatation of the blood vessels.

## Methods

### Subjects

**Figure 1.**
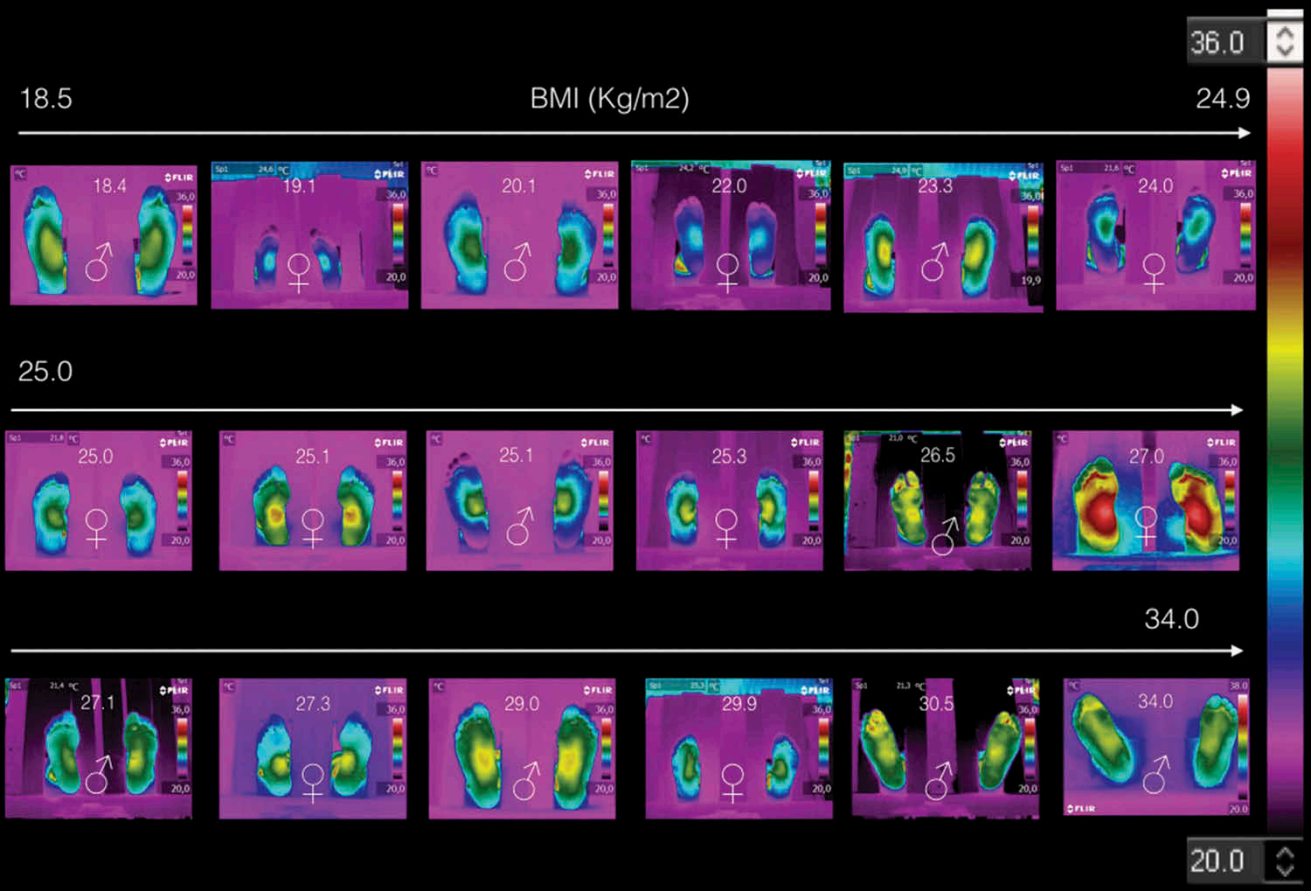
The 18 plantar skin thermograms of the volunteers, arranged as they were taken.

The thermograms of the non-diabetic volunteers were taken within the INAOE^2^ ^2^Instituto Nacional de Astrofísica, Óptica y Electrónica, is a national research facility that works together with health facilities in the country. facilities, and according to the Academy of Clinical Thermology Standards and Protocols [13]. The acquisition instruments was a Flir® E6 thermal camera. The inclusion criteria were that no signs of any sickness were presented at the acquisition time of the thermogram. The data obtained from the volunteers were the gender, age, weight and size. Table 1 summarizes the data collected; it is composed of 18 volunteers; 9 women and 9 men, BMI of 25.1 ± 3.1 kg/m^2^ and 25.6 ± 4.5 kg/m^2^ for women and men, respectively. Tables 2 and 3 show the data of those volunteers with BMI < 25 kg/m^2^ and BMI ≥ 25 kg/m^2^, respectively. The thermograms of these volunteers are displayed on Figure 1, they are arranged as a function of the BMI. Each thermogram shows the BMI and gender (by the international symbols for woman and man), at the top and bottom center, respectively. The color scale representing the temperature is show to the most right of the Figure 1.

**Table 1.** Data collected from the 18 volunteers.

Gender	Number of volunteers	Age (years)	Weight (kg)	Size (cm)	BMI* (kg/m^2^)
All	18	31.1 ± 8.0	68.0 ± 11.9	163.6 ± 10.0	25.4 ± 3.9
Women	9	33.6 ± 10.2	61.3 ± 9.7	156.1 ± 5.6	25.1 ± 3.1
Men	9	29.0 ± 5.2	73.4 ± 11.1	169.7 ± 8.5	25.6 ± 4.5

* BMI: Body mass index.

**Table 2.** Data of individuals with BMI < 25 kg/m^2^.

Gender	Number of volunteers	Age (years)	BMI (kg/m^2^)
Women	3	36.0 ± 9.5	21.7 ± 2.5
Men	3	24.8 ± 1.3	21.1 ± 2.0

**Table 3.** Data of individuals with BMI ≥ 25 kg/m^2^.

Gender	Number of volunteers	Age (years)	BMI (kg/m^2^)
Women	6	32.3 ± 11.2	26.7 ± 1.8
Men	6	31.4 ± 5.1	28.2 ± 3.2

The thermograms of the diabetic volunteers were taken within a public hospital (Hospital General del Sur, Puebla, Mexico). Since the acquisition of the thermogram is a noninvasive technique, these volunteers attended an oral presentation about the acquisition of the thermograms and the importance to have their participation in favor to prevent the diabetic complications. To those, who attended the presentation, were invited to participate. To those who agreed were given an appointment so as to satisfy with Academy of Clinical Thermology Standards and Protocols [13]. The data collected, from the diabetic volunteers, are the age, time of diabetes diagnosis (TDD), BMI, waist (*W*), and HbA1c, which averaged values are displayed in Table 6.

**Table 4.** Averaged temperatures on the medial longitudinal arc, angiosomes, and total averaged of the angiosomes for individuals with BMI < 25 kg/m^2^, for the right and left feet. LCA: lateral calcaneal artery, MCA: medial calcaneal artery, LPA: lateral plantar artery, MPA: medial plantar artery.

Gender	LCA Temp. Right (°C)	MCA Temp. Right (°C)	LPA Temp. Right (°)	MPA Temp. Right (°)	Medial longitudinal arc Right Temp. (°C)	Medial longitudinal arc Left Temp. (°C)	MPA Temp. Left (°C)	LPA Temp. Left (°)	MCA Temp. Left (°)	LCA Temp. Left (°C)	Angiosome average temp.
Women	23.8 ± 0.4	24.1 ± 0.3	23.8 ± 0.7	23.9 ± 0.6	26.2 ± 1.1	25.8 ± 0.8	23.8 ± 0.6	23.8 ± 0.8	24.1 ± 0.3	24.0 ± 0.2	24.0 ± 0.4
Men	26.4 ± 0.4	27.0 ± 0.7	26.0 ± 0.6	26.5 ± 0.7	29.0 ± 0.5	28.9 ± 0.7	26.3 ± 0.9	26.1 ± 0.7	27.0 ± 0.5	26.5 ± 0.4	26.5 ± 0.5

**Table 5.** Averaged temperatures on the medial longitudinal arc, angiosomes, and total averaged of the angiosomes for individuals with BMI ≥ 25 25 kg/m^2^, for the right and left feet.

Gender	LCA Temp. Right (°C)	MCA Temp. Right (°C)	LPA Temp. Right (°C)	MPA Temp. Right (°C)	Medial longitudinal arc Right Temp. (°C)	Medial longitudinal arc Left Temp. (°C)	MPA Temp. Left (°C)	LPA Temp. Left (°C)	MCA Temp. Left (°C)	LCA Temp. Left (°C)	Angiosome average temp.
Women	26.9 ± 1.1	27.6 ± 1.1	26.3 ± 0.6	26.6 ± 0.7	29.4 ± 1.1	29.4 ± 1.1	26.3 ± 0.6	26.0 ± 0.6	27.4 ± 1.1	26.9 ± 0.9	26.8 ± 1.2
Men	27.9 ± 2.2	28.4 ± 2.0	28.3 ± 2.4	28.7 ± 2.1	30.0 ± 1.1	30.0 ± 1.0	28.6 ± 1.8	28.2 ± 2.0	28.4 ± 1.8	28.0 ± 2.0	28.6 ± 2.5

**Table 6.** Data collected from the 40 diabetic volunteers.

Gender	Number of volunteers	Age (years)	Time of diabetes diagnosis (years)	BMI* (kg/m^2^)	Waist (cm)	HbA1c
All	40	57.0 ± 12.5	12.5 ± 8.3	30.5 ± 5.2	95.3 ± 12.8	7.5 ± 1.8
Women	37	58.1 ± 10.6	13.7 ± 8.5	30.2 ± 4.9	95.5 ± 14.1	7.3 ± 1.7
Men	3	52.1 ± 5.7	7.3 ± 4.8	32.0 ± 6.5	94.5 ± 4.2	8.5 ± 2.2

* BMI: Body mass index

## Results

### Thermograms of individuals with the BMI < 25 kg/m^2^

**Figure 2.**
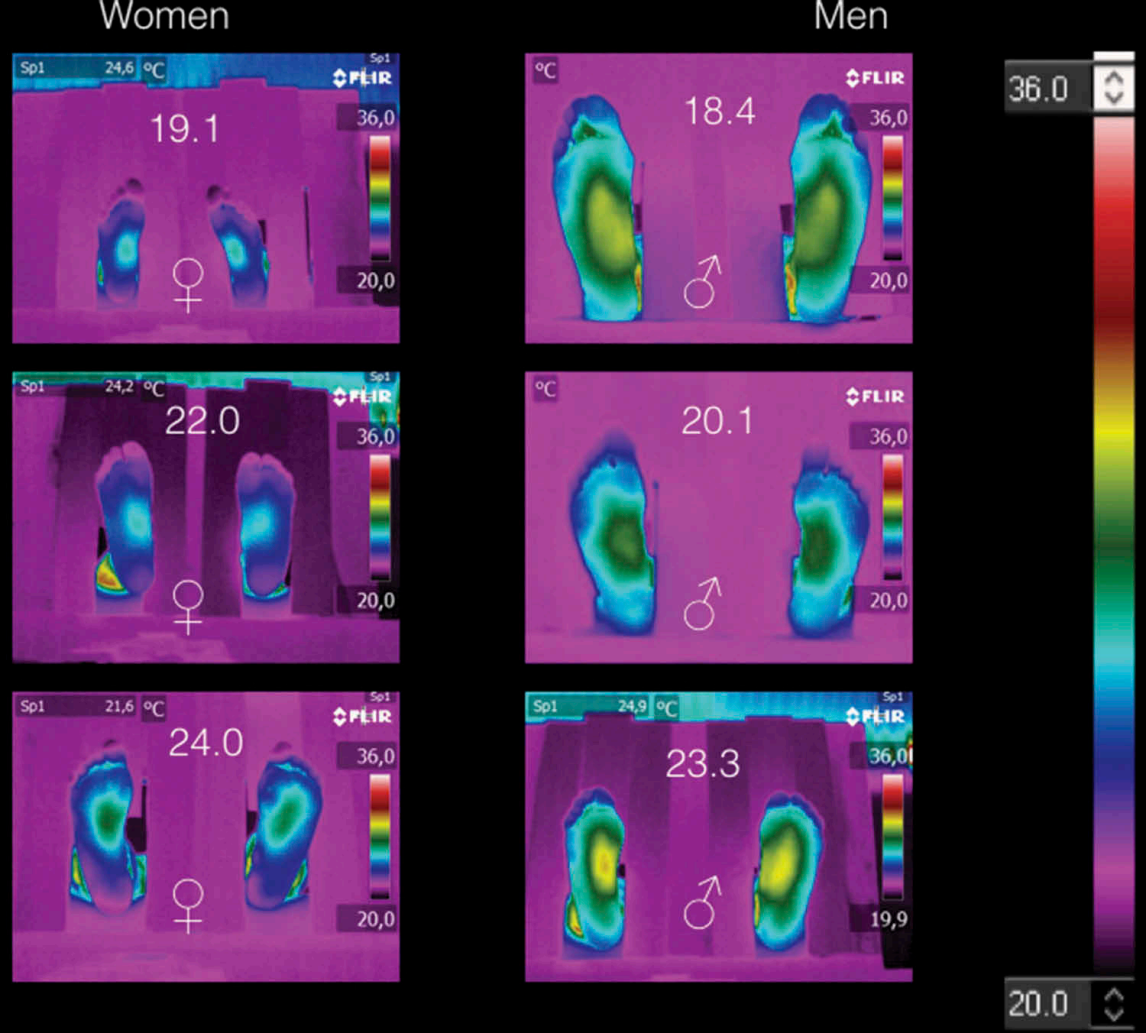
Thermograms of volunteers with BMI < 25 kg/m^2^. Three main characteristics are shown: (1) symmetry, i.e., the temperature distribution on the right feet is almost the mirror image of those of the left feet. (2) The medial longitudinal arch is the hotter part of the plantar skin for both women and men. (3) The temperature decreases distally from the medial longitudinal arch to the periphery.

Figure 2 shows the thermograms of the volunteers with BMI < 25 kg/m^2^. The thermograms arranged by gender. Table 4 shows the average temperatures of the medial longitudinal arch and the four angiosomes for the right and left feet, respectively. Thus, from Figure 2 and corroborated by Table 4, the thermograms of women are distinguished from those of men, because the averaged angiosomes temperature is almost three degrees coldest than those of the men. Furthermore, all these thermograms show three main characteristics: (1) symmetry, i.e., the temperature distribution on the right feet is almost the mirror image of those of the left feet, as can be corroborated from the corresponding angiosomes averaged temperatures (see Table 4). (2) The medial longitudinal arch is the hottest part of the plantar skin for both women and men. (3) The temperature decreases distally from the medial longitudinal arch to the periphery (fingers, heel, and lateral longitudinal arch).

### Thermograms of individuals with the BMI ≥ 25 kg/m^2^

**Figure 3.**
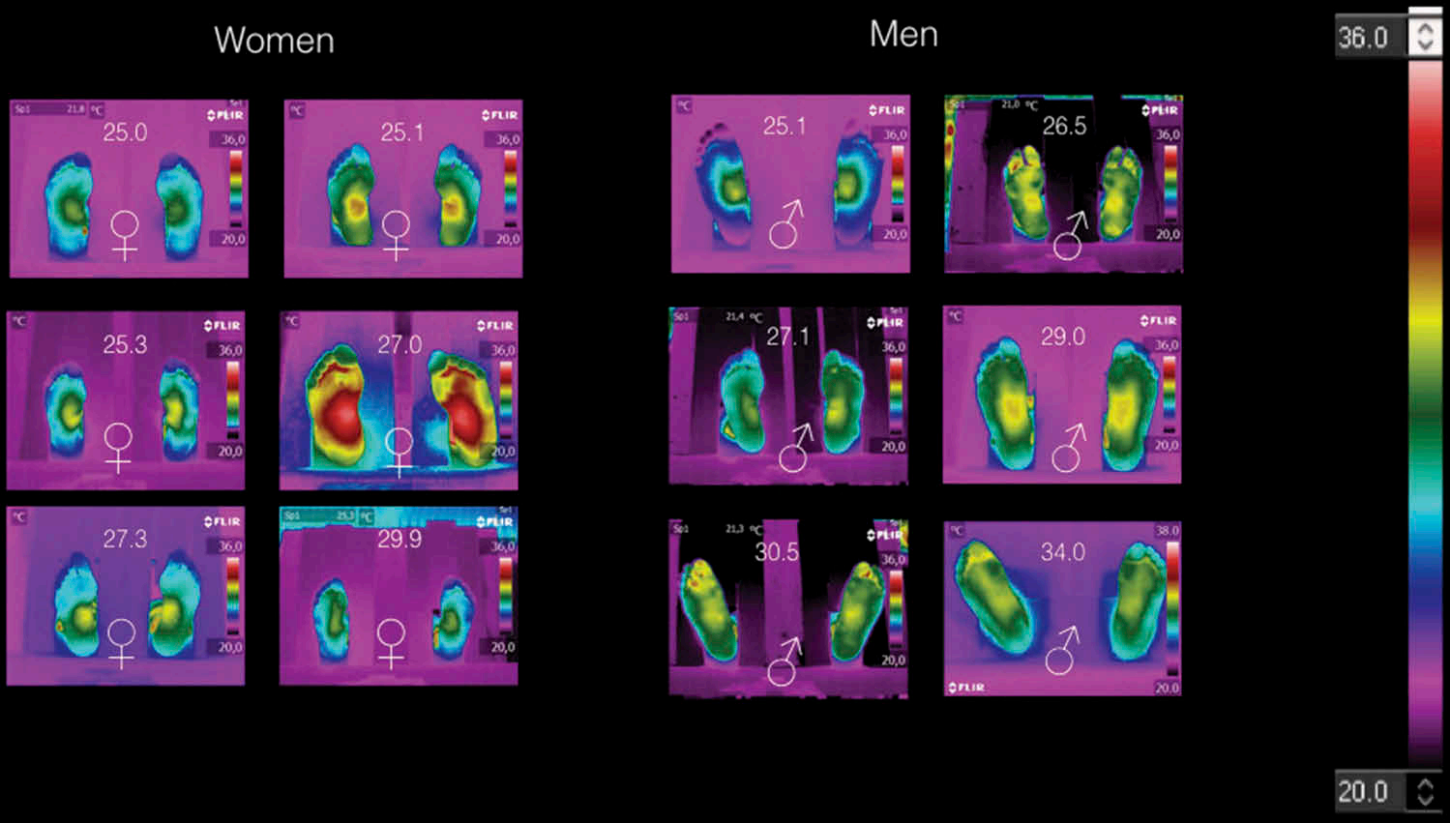
Thermograms of volunteers with BMI ≥ 25 kg/m^2^. It is not possible to distinguish women thermograms from those of men thermograms. However, symmetry is observed in some of these thermograms. The temperature distribution is still decreasing from the middle to the periphery of the foot.

Figure 3 shows the thermograms of volunteers with BMI ≥ 25 kg/m^2^, arranged by gender. Here, it is impossible to distinguish women thermograms from those of the men. Table 5 shows the average temperatures of the medial longitudinal arch and the four angiosomes for the right and left feet, respectively. Although, the symmetry is observed in some of these thermograms, but the medial longitudinal arch and the angiosomes average temperatures are almost two degree hotter than those temperatures given in Table 4. The temperature distribution is still decreasing from the middle to the periphery of the foot. However, the standard deviation, for each averaged temperature of the angiosomes, shows greater uncertainty.

### Thermograms of diabetic volunteers

**Figure 4.**
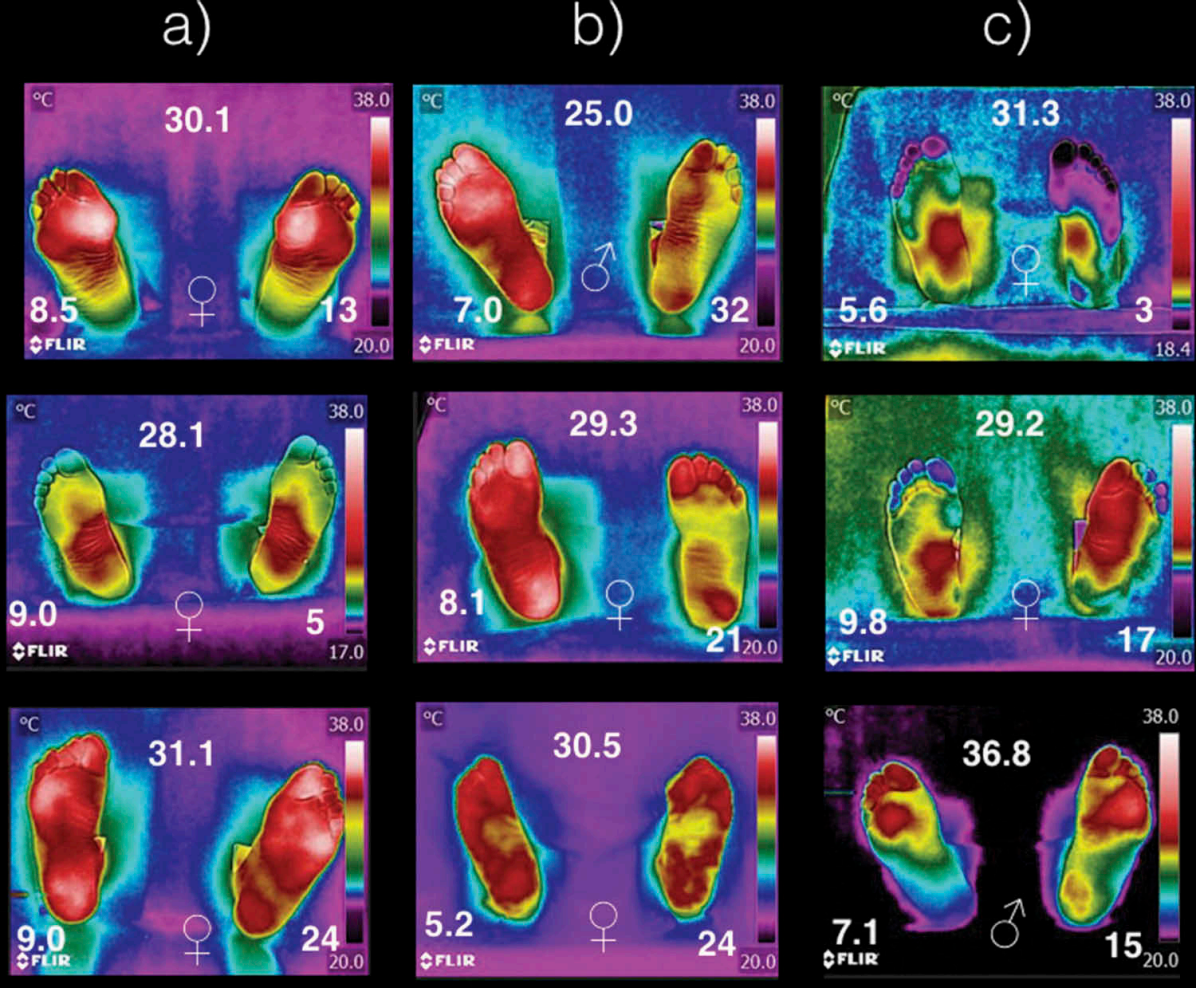
Thermograms of nine diabetic patients. The symbols ♀ and ♂ represent woman or man, respectively. The number at the top is the BMI (given in kg/m^2^). The HbA1c is shown at the left-bottom, while the time with diagnostic of diabetic is given at right-bottom (years). The columns (a), (b), and (c) represent how temperature distribution is presented in the diabetic patients.

Figure 4 shows some thermograms samples from the 40 diabetic volunteers. Within, each thermogram, it is shown the gender (center-bottom), the BMI (center-top), HbA1c (left-bottom), and the time of diabetes diagnosis (right-bottom). The temperature distribution on the plantar skin of diabetic patients can be (a) overheated and symmetric within the foot and between the both feet, (b) overheated and symmetric on one foot, and partially overheated on the other one without symmetry, and (c) asymmetric, overheated and cold on different regions on the same foot. The meaning of overheated and coldest is to have temperature distributions higher or lower than the average temperature of that given in Table 4, respectively.

## Discussion

The thermograms of Figure 2, which correspond to individuals with BMI < 25 kg/m^2^, could be addressed as the standards, i.e., how healthy individuals thermoregulate their plantar skin. This means that the physiological process, to keep or dissipate heat, is achieved properly. The fingers and the periphery of the foot are colder than the medial longitudinal arch, on both feet.

The thermograms of individuals with BMI ≥ 25 kg/m^2^, which informed us that they were not sick at the acquisition time, show a different temperature distribution as compared with the thermograms of the healthy individuals (see Figure 2). In this case, the physiological process to regulate the temperature of the plantar skin is been affected somewhere within its trajectories (that is, from the free nerve ending up to the integration structure in the hypothalamus, and/or from the hypothalamus up to the effectors to keep or dissipate heat).

The thermograms on Figure 3 show that in the overweight and obese individuals, there is an overheating on their plantar skin. Thus, the overheating of overweight and obese volunteers probes that the hypothalamus is not properly thermoregulating the plantar skin. However, the peripheral effectors, that participate in keeping or dissipating heat (vasoconstrictors, vasodilators, sweat glands, etc.), could also be altered. It may find an explanation by the fact that the excess of adipose tissue, which isolates thermally the human body, needs to dissipate more heat. However, from the thermograms on Figure 3, the asymmetric temperature distribution on the feet indicates local heat dissipation abnormalities.

**Figure 5.**
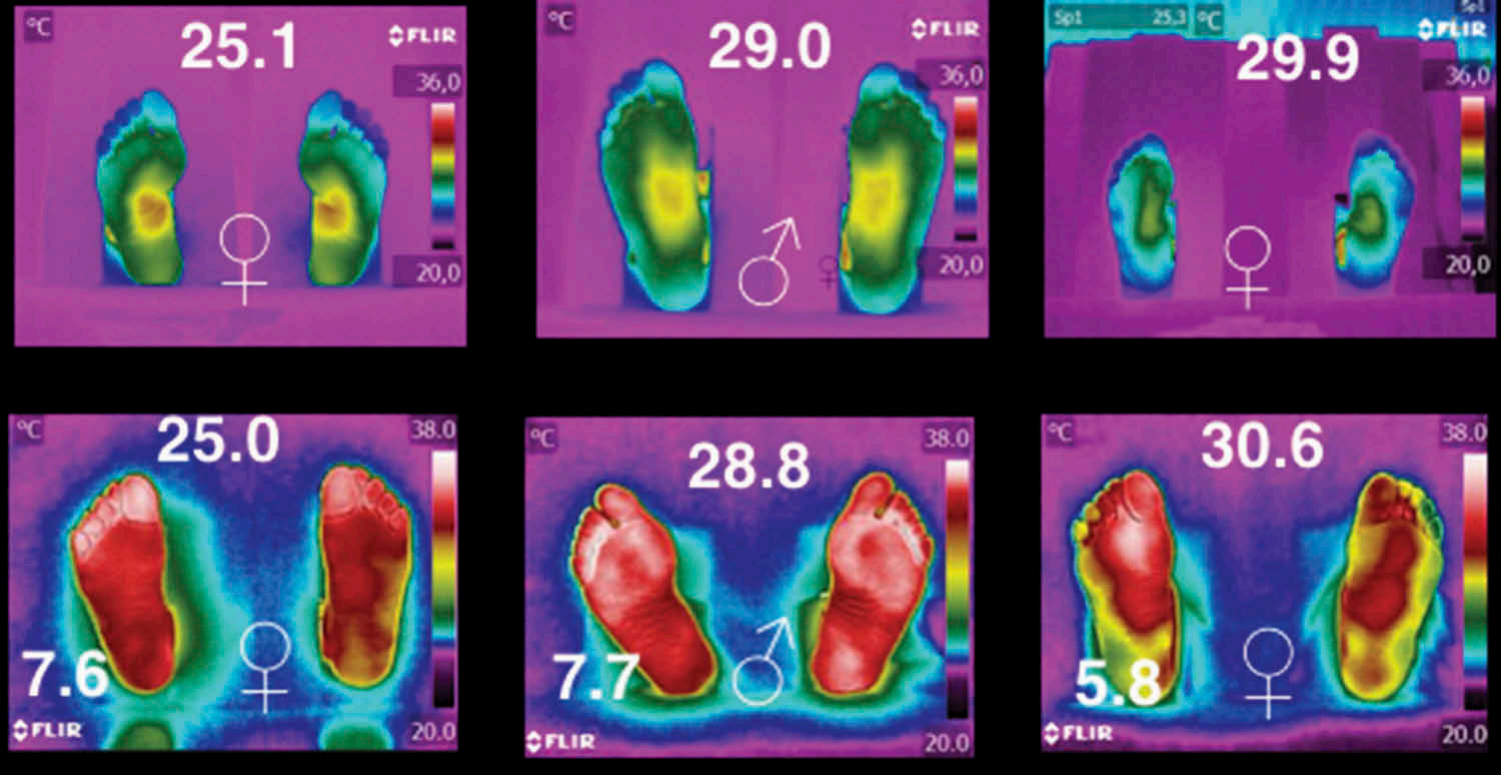
Six thermograms of the plantar skin. The three on the top corresponds to the individuals with BMI ≥ 25 kg/m^2^, while the three at the bottom are from the database of diabetic volunteers [12]. The symbols ♀ and ♂ represent woman and man, respectively. The BMI of the individual is given at the central-top of each of the thermograms (given in kg/m^2^), and the HbA1c is given at the left-bottom for those thermograms of the diabetic patients.

The six thermograms of the plantar skin, shown in Figure 5, correspond to individuals with BMI ≥ 25 kg/m^2^ (the three on the top), while the three at the bottom are from the database of diabetic volunteers [12]. Within these thermograms the symbols ♀ and ♂ represent woman and man, respectively. The BMI of the individual is given at the central-top of each of the thermograms (given in kg/m^2^), and the HbA1c is given at the left-bottom for those thermograms of the diabetic patients. The thermograms are arranged as a function of the BMI from left to right. The top and the bottom thermograms correspond to non-diabetic and diabetic individuals, with almost the same BMI, respectively. These thermograms evidence, that once the diabetes has been established, the physiological process of the thermoregulation of the plantar skin is damaged, and it seems to be not reversible.

In diabetes mellitus type 2, it had been shown that the peripheral vessels and nerves are damaged producing an irregular thermoregulation of both feet [16–18], which is easily corroborated from the thermograms on Figure 4 since they differed a lot from those thermograms shown in Figure 2. Most of the literature concludes that the feet of the diabetic patients have temperature increased [12,15]. This means, if the plantar skin of the diabetic patient is getting hot, that the vessels of that skin region are getting blood just as if needed to dissipate heat. Those regions of the plantar skin, where the temperature dropped below those temperatures shown in Table 4, are of interest. For instance, the left foot of the first thermogram of column (c) in Figure 4, shows and average temperature of 20°C on both angiosomes, MPA and LPA. On the third thermogram, in column (c) in Figure 4, the angiosomes MCA and LCA of the right foot show averaged temperatures of 22°C. That is, on the cold skin regions, less blood is passing through those vessels. The less blood passing through the vessels of the cold skin may be a consequence of the sensibility loss, or that the constriction or dilatation of the blood vessels is achieved partially. Thus the cold part of the plantar skin, on the asymmetric thermograms of diabetic patients, may be considered a risk tissue to develop more complications (trauma, infections, diabetic foot).

## Limitations of the study

The main limitation of this study, regarding the overweight and obese individuals, was the sample data, which is mainly composed of Hispanic individuals, and was obtained locally (within the institution and three hospitals). Thus, studies with different ethnic populations should be performed so as to correlate the thermoregulation with the BMI.

Since, it is well known that obesity is a risk factor to develop diabetes, thus, a specific protocol to study the thermograms of these individuals, should be conducted, so as to learn more about their thermoregulation and the risk to develop diabetes. On the other hand, the thermograms of the diabetic patients should be taken periodically, so as to look for temperature changes, within the foot and between feet, even in controlled diabetic patients. This is due to fact, that elevated glucose levels may damages the blood vessels and/or nerve system at any time.

## Conclusions

It can be observed that without sign of sickness and BMI < 25 kg/m^2^, the thermograms of the feet are symmetric (the thermogram of the right foot is the mirror image of the thermogram of the left foot). Furthermore, the medial longitudinal arch is the hottest part and its average temperature is 26.0 ± 1.0°C and 29.0 ± 0.6°C for women and men, respectively.

The thermograms of those volunteers, with BMI ≥ 25 kg/m^2^, are asymmetric, and have an increase of the temperature in the medial longitudinal arch. This irregular heating of the plantar skin may be a sign, that the CNU or the effectors, that achieves the dissipation or the preservation of heat, is/are having trouble to control plantar skin temperature.

Most of the thermograms of the diabetic patients, whatever the gender, BMI, HbA1c and the time of diagnosis, show an increase of the temperature distribution on the plantar skin on both feet. However, in the asymmetric thermograms, the cold regions of the plantar skin can be considered a tissue of risk, because of the reduction of the local autonomic sensing and the lack of achieving properly the vasodilatation. Thus, that foot of the diabetic patient may be considered of risk to develop complications (trauma, infections, diabetic foot).
